# Adverse Health Effects of Betel Quid and the Risk of Oral and Pharyngeal Cancers

**DOI:** 10.1155/2017/3904098

**Published:** 2017-12-11

**Authors:** Ping-Ho Chen, Qaisar Mahmood, Gian Luigi Mariottini, Tai-An Chiang, Ka-Wo Lee

**Affiliations:** ^1^School of Dentistry, College of Dental Medicine, Kaohsiung Medical University, No. 100 Shih-Chuan 1st Road, Kaohsiung 80708, Taiwan; ^2^Department of Medical Research, Kaohsiung Medical University Hospital, No. 100 Shih-Chuan 1st Road, Kaohsiung 80708, Taiwan; ^3^Cancer Center, Kaohsiung Medical University Hospital, Kaohsiung Medical University, No. 100 Shih-Chuan 1st Road, Kaohsiung 80708, Taiwan; ^4^Institute of Biomedical Sciences, National Sun Yat-sen University, No. 70 Lienhai Road, Kaohsiung 80424, Taiwan; ^5^Department of Environmental Sciences, COMSATS Institute of Information Technology, Abbottabad, Pakistan; ^6^Department of Earth, Environment and Life Sciences (DISTAV), University of Genova, Viale Benedetto XV 5, 16132 Genova, Italy; ^7^College of Human Science and Technology, Chung Hwa University of Medical Technology, No. 89, Wenhwa 1st St., Rende Shiang, Tainan 71703, Taiwan; ^8^Department of Otolaryngology, Kaohsiung Medical University Hospital, No. 100 Shih-Chuan 1st Road, Kaohsiung 807, Taiwan; ^9^Department of Otolaryngology, College of Medicine, Kaohsiung Medical University, No. 100 Shih-Chuan 1st Road, Kaohsiung 80708, Taiwan

## Abstract

Global reports estimate 600 million betel quid (BQ) chewers. BQ chewing has been demonstrated not only to be a risk factor for cancers of the oral cavity and pharynx and oral potentially malignant disorders (OPMD) but also to cause other cancers and adverse health effects. Herein, we summarized the international comparison data to aid in the understanding of the close relationship between the prevalence of BQ chewing, the occurrence of oral and pharyngeal cancers, and adverse health effects. Potential biomarkers of BQ carcinogens, such as areca nut, alkaloids, and 3-methylnitrosaminopropionitrile (MNPN), are closely associated with human health toxicology. Molecular mechanisms or pathways involving autophagy, hypoxia, COX-2, NF-*κ*B activity, and stemness are known to be induced by BQ ingredients and are very closely related to the carcinogenesis of cancers of oral and pharynx. BQ abuse-related monoamine oxidase (MAO) gene was associated with the occurrence and progress of oral and pharyngeal cancers. In summary, our review article provides important insights into the potential roles of environmental BQ (specific alkaloid biomarkers and nitrosamine products MNPN) and genetic factors (MAO) and offers a basis for studies aiming to reduce or eliminate BQ-related OPMD and oral/pharyngeal cancer incidences in the future.

## 1. Introduction

Betel quid (BQ) is an environmental carcinogen with human health toxicology. In Asia and among diverse migrant populations in western countries, BQ use is an emerging health-related issue. In the world, BQ chewing is the fourth most common psychoactive habit after the usage of tobacco, alcohol, and caffeine beverages [1]. It is a masticatory mixture containing various components, such as areca nut (AN), slaked lime (calcium hydroxide), betel leaf, and locally varied flavorings [2]. In many countries, integration of tobacco, another known carcinogen, into the BQ is practiced [2]. It has been reported that 600 million chewers worldwide (approximately 10% of the population) use certain variety of BQ, mainly in South East and South Asia, in the Indo-Pakistan subcontinent, in mainland China (Hunan Province and Hainan Special Administrative Region), in Taiwan, and on the South Pacific region (such as Palau, Papua New Guinea, and Solomon Islands) [3, 4]. Moreover, BQ chewing is also common among Asian immigrants to the Africa, Australia, United States, and United Kingdom [2, 3, 5].

Users chew BQ for its pharmacological effects, such as well-being sensations and euphoria, heightened alertness, and focused attention, as well as diminished hunger and improved digestion [6, 7]. BQ is not only a psychostimulant and addictive substance [8] but also a carcinogen [9]. The International Agency for Research on Cancer (IARC) disclosed that BQ substances, with and without tobacco additives, have been classified as group I carcinogens in humans, and elevated risks were noted for oral [10, 11] and pharyngeal [12] cancers and oral potentially malignant disorders (OPMD) [13, 14]. In addition, AN is also a group I carcinogen for humans [9]. In animal experiments, there is strong evidence suggesting that BQ can induce the occurrence of cancers of oral and pharynx [9].

Habitual BQ chewing has been especially related to the occurrence and development of OPMD [13, 14, 18] and oral/pharyngeal cancers [10, 11, 19]. In many areas, tobacco-free BQ, commonly in conjunction with the usage of tobacco and/or alcohol, induces early cancer occurrence for specific upper aerodigestive tract cancers and affects the incidence pattern of tumor site of these neoplasms [20]. In addition, epidemiological investigations have revealed that prolonged BQ use confers an increased risk of esophagus [21], hepatocellular carcinoma (HCC) [22–24], liver cirrhosis [25], metabolic syndromes [26, 27], likely contributing to type 2 diabetes mellitus (DM) [28], high blood pressure [29], cardiovascular disease [30, 31], heart disease in women [32], chronic kidney disease [33], and adverse effects on mortality from cancer and from all causes [30, 34, 35]. BQ addiction has also been linked to anemia [36], adverse pregnancy outcomes [37, 38], and acute severe asthma [39, 40]. Increasingly, BQ usage is recognized for its association with multidimensional health effects (Table 1).

## 2. BQ Usage Multidimensional Health Effects (Table 1)

To date, BQ chewing is associated with malignant and premalignant lesions of the oral cavity as well as other malignancies. In a case-control study, the highest risk of calculated incidence for oral cancer among BQ chewers with both smoking and drinking habits was 123-fold (95% CI 17.1–880.5) compared with abstainers [10]. This study also indicated that BQ chewing alone was an independent risk factor related to the oral cancer occurrence [10]. A further study suggested that BQ use was an important risk factor of both oral submucosal fibrosis (OSF) and oral leukoplakia [18] and smoking had a modifying effect in oral leukoplakia development [18]. In Taiwan, a case-control study of pharyngeal and laryngeal cancers demonstrated that BQ chewing habit was a prominent risk factor for the occurrence of pharyngeal cancer (adjusted odds ratio [aOR] = 7.7; 95% CI = 4.1–15.0) but not for laryngeal cancer, providing the first insight into BQ chewing effects on other digestive tract sites [12]. Investigations into esophageal cancer in one hospital-based study of 165 cases and 255 controls showed that those who chewed AN with betel inflorescence (aOR = 4.2; 95% CI = 1.4–16.0) and chewers with a habit of swallowing BQ juice (aOR = 3.3; 95% CI = 1.3–9.3) had a significantly elevated risk of esophageal cancer [21].

Another malignancy linked to BQ chewing is hepatocellular carcinoma (HCC). In case-control studies, BQ chewing was significantly associated with HCC that showed there was an additive effect between BQ use and chronic infection with either hepatitis B virus (HBV) or hepatitis C virus (HCV) as risk factors of the cancer [22–25]. A community-based cohort study indicated that BQ chewing habits were associated with the increased risk of HCC, and BQ chewers with hepatitis surface antigen- (HBsAg-) positive had the highest risk of HCC (aOR = 19.5; 95% CI = 8.7–43.4) compared with HBsAg-positive subjects without BQ chewing habits and HBsAg-negative BQ chewers [41]. Among HBsAg-seronegative subjects, the adjusted relative risk (aRR) of HCC was significantly higher for BQ chewers compared with nonchewers (aRR = 3.4; 95% CI = 1.2–9.9). In a case-control study, the habit of BQ use as a risk factor related to the development of HCC has been reported (aOR = 3.49; 95% CI = 1.74–6.96) [22]. Moreover, a higher risk of HCC was related to a longer period of BQ use and a larger quantity of BQ chewed [22, 23]; BQ chewing habit was associated with the occurrence of liver cirrhosis (aOR = 3.56; 95% CI = 1.41–8.96) [25]. A recent study demonstrated that habitual BQ chewers, with or without chronic infection of HBV/HCV, were associated with a higher risk for the occurrence of HCC (aOR = 3.73; 95% CI = 1.63–8.53) [24].

Studies on the health effects of BQ chewing have expanded to include hypertension [29], obesity [43], metabolic syndrome [26, 27], hyperglycemia [26], and hypertriacylglycerolemia [26], where the daily rate of BQ chewing was positively and independently related to these conditions among adults [27]. A population-based study on men further demonstrated an independently predictive dose-response relationship of BQ chewing on the development of metabolic syndrome [26]. Indeed, BQ chewing habit was significantly associated with type 2 DM (aOR = 1.29; 95% CI = 1.04–1.60) in men [28]. Our previous findings showed the prevalence of BQ chewing to be 46.1%, with this practice being closely related to obesity (OR = 1.61; 95% CI: 1.40–1.85). Another study found that daily chewing of BQ was independently related to heart disease in women, with a risk associated with BQ mean chewing rate of 10 times per day (aOR = 1.37; 95% CI = 1.1–1.6; *p* = 0.003) [32]. Additionally, in a Bangladesh cohort study, BQ chewers without tobacco usage had a significant relationship to systolic hypertension (aOR = 1.55; 95% CI 1.01–2.37) and hypertension (OR = 1.48; 95% CI 1.04–2.10) after controlling for potential founding variables [29]. Also, data showed that past BQ users were significantly more likely to die from cancer (adjusted hazard ratios [aHR] = 1.55; 95% CI: 1.09–2.22) and all causes (aHR: 1.26; 95% CI: 1.09–1.44) after adjusting for confounding factors, implicating BQ chewing as significantly associated with mortality from cancers and all causes [34].

Particularly, BQ use behaviors might be associated with schizophrenia in Palau [46, 47] and Sri Lanka [78]. In Palau, BQ chewers were associated with mild schizophrenia and have a benefit on patients with respect to a decrease of both positive and negative symptoms [46, 47]. However, these associations were not statistically significant in symptoms between BQ users and nonusers in Nepal [79]. The habit of BQ chewing has also been studied for adverse pregnancy outcomes. In a study of 62 women who had adverse effect on pregnancy outcome and 124 age-matched women (control group), the prevalence of substance use in aboriginal women was 43.6% for alcohol, 43.6% for BQ chewing, and 14.5% for cigarette smoking, whereas it was alcohol, 38.7%; BQ chewing, 28.2%; and cigarette smoking, 8.1% in the matched comparison group [42]. The risk of adverse pregnancy effects was 2.8-fold higher among maternal BQ use as compared with non-BQ users (aOR = 2.8; 95% CI = 1.2–6.8) [42]. A further study of 229 aboriginal women showed that an estimated risk of harmful birth outcome was statistically significantly higher among women with the habits of BQ use as compared with nonusers (aOR = 5; 95% CI = 1.1–23.0) [38]. In another study in Taiwan aborigines that included 1264 postpartum aboriginal women who used BQ during gestation were significantly related to both lower birth weight (LBW) (−89.54 g) and lower birth length (−0.43 cm) [37]. Thus, women who had chewed BQ throughout pregnancy conveyed a 2.40- (95% CI = 1.21–4.80) and 3.67-fold (95% CI = 1.70–7.96) independent risk effects for LBW and full-term LBW on gestation, respectively, and chewers were more likely to have female newborn than nonchewers [37]. Indeed, a significantly lower rate of male newborns at birth (aOR = 0.62; 95% CI = 0.43–0.89) was also observed to be associated with BQ use alone during gestation [37]. A previous animal study has indicated the influence of areca nut extract (ANE) on male reproduction. Male rats were given ANE by gavage to characterize reproductive toxicity resulting from ANE exposure [80].

## 3. Prevalence of BQ Chewing among Adults and Selected Hyperendemic Countries for Oral and Pharyngeal Cancers (Table 2)

Using data from the GLOBOCAN 2012 statistical database of estimated cancer incidence, mortality, and prevalence worldwide 7 [76], we summarized the international comparisons between the prevalence of BQ chewing and oral/pharyngeal cancer incidences (Table 2). We can identify a positive trend between oral and pharyngeal cancer incidence and BQ chewing rates in different countries. Populations with high chewing rates often have a higher incidence rate of oral and pharyngeal cancers than other countries. In BQ endemic areas, a close association is observed between a higher prevalence of BQ chewing and the age standardized rate adjusted by the world population (ASRW) for the incidence/mortality rate of oral and pharynx cancers.

In some countries (e.g., Malaysia [9] and Thailand [49]), the intermediate proportion of BQ use may result in an intermediate incidence of oral and pharyngeal cancers. There are no BQ chewing habits in some countries, such as Singapore, Japan, and Korean. Occurrences of oral and pharyngeal cancers might also be due to cigarette or alcohol consumption [9]. Thus, in most countries, BQ chewing has a modifying effect or causes an interaction with the practices of cigarette or alcohol to induce oral and pharyngeal cancers. However, there is a close relationship between oral/pharyngeal cancers and the higher prevalence of BQ use in endemic BQ chewing areas.

### 3.1. Taiwan

In Taiwan, the incidence (2012 ASRW) adjusted by the 2000 world-standard population for oral and pharyngeal cancers was 41.7 per 100,000 among men and 3.5 per 100,000 among women [77]. According to the latest 2014 Taiwan Cancer Registry data, the incidence and mortality rates (the rates were adjusted by world population in 2000) of men with cancers of oral and pharynx are as high as 42.85 and 15.41 per 100,000 male population, respectively [81]. According to the IARC, Taiwan reported a 44-fold increase in AN production between 1961 and 2001 [9], which could lead to a rapid growth in oral and pharyngeal cancer incidences in the future. Indeed, from 2007 to 2014, there were more than 5,000 new cases of men with the cancers of oral and pharynx each year and this has continued to increase to 7,600 cases in Taiwan [82]. In the past 10 years (2004–2014), the incidence and mortality rates of oral and pharyngeal cancers for men were stable elevation and ranked fourth in cancer occurrence and deaths [82].

In a 2007 published international comparison, the fourth highest incidence of cancers of oral and pharynx among males was ranked in Taiwan, followed only by Papua New Guinea, the Solomon Islands, and Sri Lanka [4]. Oral and pharyngeal cancers are prevalent in Taiwan, particularly in male BQ chewers. Correspondingly, previous studies indicated that the prevalence of BQ chewers is 14.5%–16.5% in men [48, 67, 69]. There are two million habitual BQ chewers in Taiwan alone (approximately 10% of this island's people) [67]. BQ is usually practiced by men (men: 16.5%; women: 2.9%) [67], lower education level, blue-collar job, subjects with smoking habits, alcohol drinkers, and Taiwan aborigines. A national survey indicated that men had a higher rate of BQ chewing than women (men: 20.9%; women: 1.2%) [70]. In this survey, the highest rate of BQ chewing was among Taiwan aborigines: 54.3% of men and 33.8% of women [70]. However, another aboriginal study revealed that current chewing habits were more common among women (78.7%) than men (60.6%) [83]. In national health study, the lifetime chewing rates (combined analysis of current-chewers and ex-chewers) were also found to be relative higher in men (18.9%) as compared to women (1.7%) [68].

**Figure 1:**
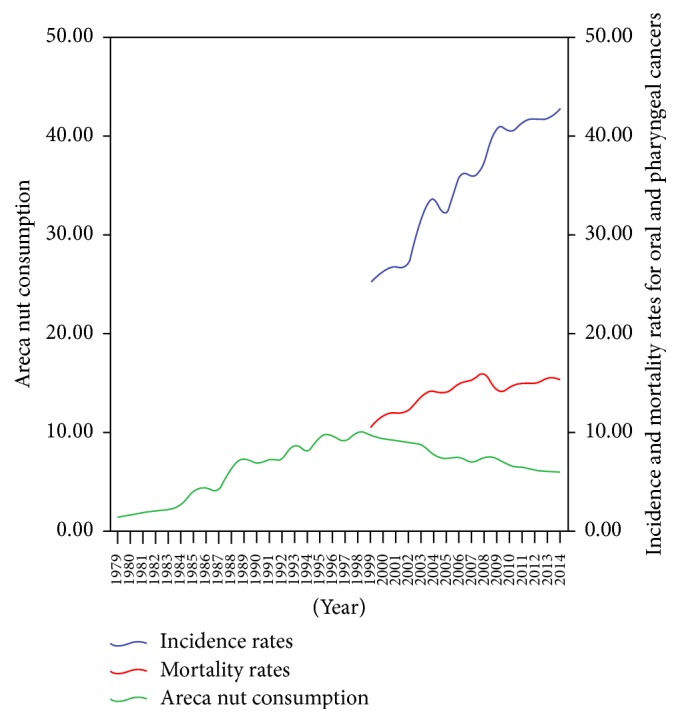
The long-term trend of incidence and mortality (ASRW) due to oral and pharyngeal cancers among males (per 100,000 population) and areca nut consumption (kg) of per person (population of 15 years) per year in Taiwan.

Recently, an intercountry survey of Asian betel-quid consortium also revealed that the proportion of BQ chewing among Taiwanese men (15.6%) was significantly higher than that among Taiwanese women (3.0%) [48]. Currently, there are no legal age restrictions that must be strictly observed for BQ use. In adolescents, the proportion of BQ was practiced by 31.0% of the vocational school students (36.1% for boys; 8.3% for girls) compared with 14.0% of the junior high school students (24.4% for boys; 5.0% for girls) [84]. As shown in Figure 1, the green line is the estimated per person per year areca nut (AN) consumption (kg); blue lines and red lines are age-standardized rates of incidence and mortality for oral and pharyngeal cancers (per 100,000 men population) showing the long-term trend (1999–2014). It is noteworthy that the incidence and mortality rates of oral and pharyngeal cancers showed a steady upward trend from 1999 to 2014. In Taiwan, if we cannot propose an effective preventive and controlling strategy for BQ-related oral and pharyngeal cancers; this situation will continue to deteriorate, and oral and pharyngeal cancer incidences will soon be the highest in the world. Therefore, control and prevention strategies of the occurrence of oral and pharyngeal cancers are an indispensable issue.

Almost 80% of oral and pharyngeal patients have BQ chewing habits and epidemiological studies suggest that BQ use significantly elevates the risks of oral and pharyngeal cancers [10, 11, 19]. Compared with no-chewers, BQ chewers will develop oral and pharyngeal cancers 10 years earlier, with lower 5-year survival rates and their oral mucosal lesions are 8.21 times larger than nonchewers [85]. Therefore, BQ chewing habits are closely related to the occurrence and prognosis of oral and pharyngeal cancers. In regard to BQ-related oral and pharyngeal cancer survival, we reported that Taiwanese men have a high prevalence of BQ use and this habit is related to poor survival rates observed [86]. The 5-year survival rates of oral and pharyngeal cancers are significantly lower for the community of Hokkien people (Han Chinese) (53.9%) and the Taiwanese aborigines (58.1%) than Hakka community (60.5%). Genetic predispositions and lifestyle habits (e.g., BQ chewing, cigarette smoking, and alcohol drinking) were considered as crucial factors in the differences of survival among racial groups with respect to oral and pharyngeal cancers [87].

### 3.2. Papua New Guinea

In the population of Papua New Guinea, the incidence (2012 ASRW) of oral and pharyngeal cancers among men was 34.8 per 100,000 and 21.7 per 100,000 among women. Also, the rate of BQ use was higher (57.7%) in Papua New Guinea, 62.8% for men and 52.8% for women [72]. However, the Papua New Guinea statistical data are from nearly 49 years ago and conceivably this is necessary to be reinvestigated. Two relatively new studies also reported very high rates of lifetime chewing (76% and 93.1%, resp.) [73, 74].

### 3.3. Bangladesh

In Bangladesh, the incidence (2012 ASRW) of oral and pharyngeal cancers in men was 27.9 per 100,000 and 9.0 per 100,000 in women. A prospective population-based study (19,934 Bangladeshi adults) found that the proportion of current BQ chewers was 33.2% in this cohort (35.5% of men and 31.6% of women, resp.); the lifetime chewers were 34.9% (38.4% of men and 32.5% of women, resp.) [29].

### 3.4. Sri Lanka

The estimated incidence (2012 ASRW) of oral and pharyngeal cancers was still higher in Sri Lanka and the rate in men was 22.7 per 100,000 and 9.0 per 100,000 in women. In a large-scale survey, the proportion of BQ chewers was 45.2%, 54.0% for men and 42.0% for women, respectively [66]. More recently, an international survey by Asian BQ Consortium study indicated that the prevalence of BQ chewers was only 16.9% (21.2% for men and 14.5% for women) [48].

### 3.5. Myanmar

The oral and pharyngeal cancer incidences (2012 ASRW) were calculated to be 17.7 per 100,000 among men and 5.8 per 100,000 among women in the Myanmar population. An early report indicated that 24.5% were BQ chewers (combine 16.2% regular chewers with 8.3% occasional chewers) [51]. Recently, a higher chewing rate was found in Dagon Myothit (East) Township, Myanmar; there, 52.4% of subjects indulged in the habit (72.4% for men and 38.6% for women) [52].

### 3.6. India

In India, the 2012 ASRW for oral and pharyngeal cancer incidences was also noted to be high (16.4 per 100,000 for men and 5.6 per 100,000 for women). India has the largest population of BQ consumption worldwide. In Mumbai (Bombay), India, a previous large-scale survey reported that 33.0% chewers used BQ in all forms, 37.8% among men and 29.7% among women, respectively [60]. However, only 0.4% of men and 0.5% of women used AN without tobacco [60]. A survey on AN habits and tobacco was conducted in the Global Adult Tobacco Survey (GATS) of India [61]. This survey reported that 6.2% subjects (men, 7.5%; women, 4.9%) chewed BQ with tobacco; 13.1% of the men and 2.9% of the women added tobacco and lime to the* Gutka* and other areca nut mixtures [61]. In addition, the prevalence of* Pan masala* or BQ without tobacco was 3.5% for men and 5.4% for women [61].

### 3.7. Pakistan

In Pakistan, the incidence (2012 ASRW) of oral and pharyngeal cancers was 13.5 per 100,000 men and 13.2 per 100,000 women. There was a high proportion of BQ use and an elevated incidence rate of cancers of oral and pharynx in Pakistan. In 1974, there were 32.5% subjects currently in the habit of chewing BQ (27.9% for men and 37.8% for women) [62]. A few previous studies indicated that the prevalence of AN use among adults was 32%, 37.8%, and 40%, respectively [63–65].

### 3.8. Nepal

The 2012 ASRW for oral and pharyngeal cancer incidence was also higher (11.1 per 100,000 for men and 3.1 per 100,000 for women) in Nepal. Recently, the national population-based survey reported that the prevalence of BQ chewers was 40.7% (43.6% for men and 34.9% for women) [48].

### 3.9. Cambodia

In Cambodia, the 2012 ASRW for oral and pharyngeal cancer incidence was 10.9/100,000 per annum for men and 6.3/100,000 per annum for women. Moreover, an earlier report indicated that 31.2% subjects were habitual BQ chewers (6.8% for men and 40.6% for women) [58]. Recently, in a population-based study conducted among Cambodia adults, 19.7% of women indulged in the BQ habits [59]. In addition, the highest risk of OPMD was associated with BQ chewing habits [59].

### 3.10. Thailand

In Thailand, the incidence (2012 ASRW) of oral and pharyngeal cancers was 7.7/100,000 per annum for men and 3.4/100,000 per annum for women. In northern Thailand, a previous study reported only 2.6% of subjects were occasional BQ users, and 6.8% chewed it daily. Daily BQ chewers were most prevalent in the group aged 50 years and above (22.7% of women and 18.4% of men) [88]. The BQ chewing habit seems to be on the decline from educational campaigns since the early 1955, and this habit has been indicated more popular in the older population [89]. The Global Adult Tobacco Survey (2011) illustrated that the proportion of current use of BQ with tobacco was 0.3% among men and 3.3% among women in Thailand [50].

### 3.11. Solomon Islands

The GLOBOCAN 2012 statistical database showed that the ASRW of oral and pharyngeal cancers dramatically decreased at 6.5/100,000 per annum for men and 1.5/100,000 per annum for women, but the 5-year prevalence rate per 100,000 was still maintained at 26.0 for men and 4.2 for women [90]. It was noteworthy that the incidence (2002 ASRW) of oral and pharyngeal cancers in the Solomon Islands was very high at 37.0 per 100,000 men and 22.5 per 100,000 women from GLOBOCAN 2002 statistical database [91]. In our previous Solomon Islands study, BQ chewing was very popular in both men (83%) and women (68%) [4].

### 3.12. Malaysia

In Malaysia, the incidence for oral and pharyngeal cancers was 5.0/100,000 per annum for men and 3.4/100,000 per annum for women in 2012. The BQ chewing rate was more prevalent among women [56]. Previous studies were conducted in Sarawak, Malaysia, and showed that chewing rates among indigenous women (61.2–63.5%) were significantly higher than men (25.2–30.3%) [54, 55]. Indeed, in 2011, an intercountry Asia BQ study indicated that the habit pf BQ chewing was more common in women (32.1%) than in men (10.3%) [48].

### 3.13. Indonesia

The 2012 ASRW for oral and pharyngeal cancers per 100,000 population was 4.2 among men and 2.4 among women in Indonesia. An intercountry Asian BQ survey demonstrated that the habit of BQ chewing was more popular among women (47.8) than among men (12.4%) [48].

### 3.14. Mainland China

In China, the incidence (2012 ASRW) of cancers of oral and pharynx was 2.2 per 100,000 men and 1.0 per 100,000 women. The BQ chewing habit is common only in Hunan, Hainan, and Yunnan. A previous study was conducted to evaluate the relationships between BQ usage and OSF in Xiangtan City, Hunan Province [71]. A total of 11,046 individuals were examined, and the proportion of lifetime chewing was 39.4% for men and 30.5% for women, respectively [71]. However, in a recent epidemiological study, the lifetime chewing rates among men (29.0%) were statistically prominently higher than that among women (2.3%) in Hunan. In the 1980s and 1990s, a review article indicated that the proportion of BQ chewers in Xiangtan and Changsha of Hunan was between 64.5% and 82.7% [92].

## 4. What Are the Main Chemically Carcinogenic Substances of BQ That Induce Oral and Pharyngeal Cancers?

The composition of BQ ingredients varies greatly depending on the way they are used in different areas. However, AN is the basic constituent of a variety of widely practiced BQ, and its main constituents are BQ alkaloids and polyphenols that might be associated with the cancers of oral and pharynx.

### 4.1. Overview of the Carcinogenesis of Arecoline and Arecaidine

Most evidence demonstrates that areca alkaloids (arecoline and arecaidine) are the major causes of toxicity from AN. The IARC points out that arecoline has limited evidence for carcinogenicity in animal experiments and arecaidine has inadequate evidence in animal experiments [9]. In mammalian cells, arecoline and arecaidine can cause bacterial mutagenicity, and* in vitro *and* in vivo* tests can result in the exchange of sister chromatid, aberrations of chromosome, and the formation of micronuclei [9]. In addition to the ANE, arecoline also causes dysregulation of oral epithelial cells, leading to cell cycle arrest [93]. Arecoline and ANE can repress the growth of different oral cells (such as oral mucosa fibroblasts, gingival fibroblasts, and keratinocytes) and induce genotoxicity [94–98]. Studies of its chemical components have indicated that AN contains 11–26% tannins (a category of stimulant) and 0.15–0.67% alkaloids (a major component of which is arecoline) [5, 99]. Among these components, arecoline has a chemical structure similar to that of nicotine [9].

### 4.2. BQ Toxicology Research

#### 4.2.1. Ultramicro-Mass Analysis of the Internal Dose and Effective Dose of Toxicants

Although little is known about the disposition and metabolism of areca alkaloids (arecoline and arecaidine) and 8-hydroxy-2-deoxyguanosine (8-OH-dG) in humans, we conducted an accurate and sensitive technique to explore the exposure of toxic substances from BQ consumption [15]. Using a method of liquid chromatography-tandem mass spectrometry (LC-MS/MS), given the cytotoxic effects of arecoline and arecaidine, its direct quantification from a chewing habit was determined by [15]. The average daily amount (quids/day) shows a significant association with both arecoline (*r* = 0.52) and arecaidine levels (*r* = 0.51). This means the blood measurements of these two alkaloids are good indicators for recent BQ use. We believe that arecoline and arecaidine of blood plasma are appropriately biological indicators for short-term or immediate exposure of BQ. In blood plasma, arecaidine is a better clinical marker of BQ exposure than arecoline. In contrast to urinary 8-OH-dG, which is known to have risks on human, it is not significantly correlated to BQ exposure. We have been able to determine the levels of toxicants with ultramicro analysis* in vivo*. Once ingested, BQ is metabolized and its metabolites, such as arecoline, arecaidine, and others, may exist only at nanogram concentrations (ng/mL). After chewing inverted AN (pinang-wang BQ), we also reported that high concentrations of arecoline and arecaidine may cause ventricular fibrillation [100].

**Figure 2:**
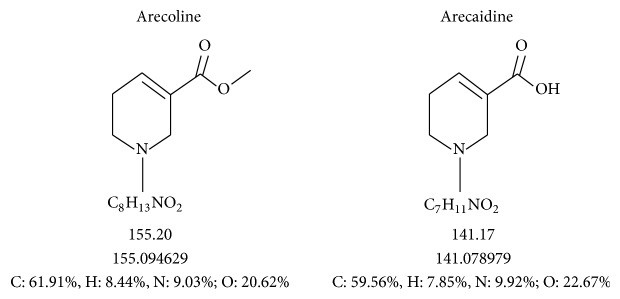
Chemical structure of major areca alkaloids [15].

BQ alkaloids let BQ chewers feel a refreshing, exciting, and warm feeling. Arecoline is the main alkaloid ingredient in BQ followed by arecaidine. Arecoline is hydrolyzed into arecaidine in the mouth of chewers. Its chemical structure is shown in Figure 2 [15]. Following human exposure, we determined the main BQ alkaloids, arecoline and arecaidine, by quantitative and qualitative analysis through LC-MS/MS. We found that arecoline and arecaidine (7.0 ± 10.7 ng/mL and 142.8 ± 249.3 ng/mL, resp.) levels in the BQ chewers' blood were significantly higher than nonchewers [15]. In addition, we found that chewed BQ quids per day and the amount of BQ chewing before drawing blood from the patient were significantly positively related to the concentration of arecoline or arecaidine in human blood [15].

### 4.3. AN Polyphenols Produce Reactive Oxygen Species

The major substances contained in the areca nut are polyphenolic compounds (tannins and flavonols), alkaloids, carbohydrates, fats, proteins, crude fiber, and minerals. A large proportion of AN dry weight is polyphenols that result in the astringent taste of AN [9]. Hence, in order to eliminate this astringency, the traders often add lime to the BQ. The polyphenols and tannins in AN play dual roles through both carcinogenetic and anticarcinogenic effects [98]. In BQ-induced tumors, AN polyphenols and tannin fractions are considered potential carcinogens [98]. However, results of many short-term experiments assays have indicated that the tannins and polyphenols of AN are not mutagenic, and conversely, they are antimutagenic. For example, some reports indicate that polyphenols can conjugate with carcinogens to bind ROS and nitrite. Conversely, a series of studies have demonstrated that the interaction between AN polyphenols and lime is the major determinant in the generation of reactive oxygen species (ROS) such as hydroxyl radical (HO^*∙*^) during BQ chewing [101–105].

Although the lime itself is not mutagenic, it will make the oral environment alkaline. The polyphenolic ingredients mixed with lime in BQ produce ROS, such as HO^*∙*^ in alkaline conditions (pH ≥ 9.5) [106]. The lime added to the BQ is indispensable to the formation of ROS and forms a hydroxyl group via the transition metal-catalyzed Haber-Weiss or via the Fenton reaction to further form the 8-OH-dG that can destroy DNA. In vitro studies have indicated that the HO^*∙*^ generation is enhanced by the catalysis of metal ions such as Cu^2+^ and Fe^2+^, and possible oxidative DNA damage is due to the generation of 8-OH-dG [102, 104, 107]. This genetic damage has been associated with oral cancer [101–104, 107, 108]. In the saliva of BQ chewers, ROS produced from AN polyphenols is crucial to initiate and promote the development of oral and pharyngeal cancers [98]. Our previous study found that chewing BQ with inflorescence of Piper betle* Linn. *(IPB) produced significantly higher amounts of HO^*∙*^ than chewing BQ with Piper betle leaf (PBL) and may induce larger oxidative stress damage to the cells of oral mucosa [109]. If this trend is repeated, long-term accumulation in the body will cause oxidative damage to the cells [110]. Epidemiological studies also support that ROS might be responsible for the possible mechanism of the oral malignant tumor process [111], and we further assume that active oxygen species may play a crucial role in BQ-related oral and pharyngeal cancers [106].

### 4.4. Overview of Carcinogenicity of Nitroso Compounds in AN

**Figure 3:**
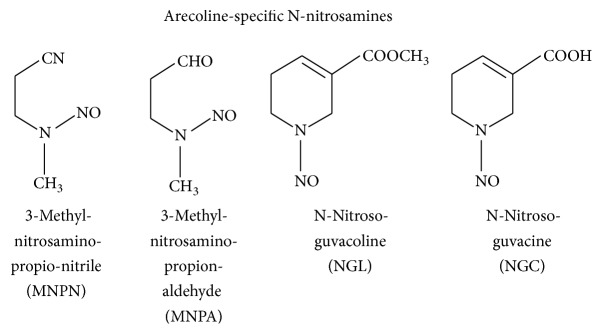
Arecoline can be formed as AN-specific nitrosamine substances (areca-specific N-nitrosamines) by nitrosation reaction in the human body [16].

**Figure 4:**
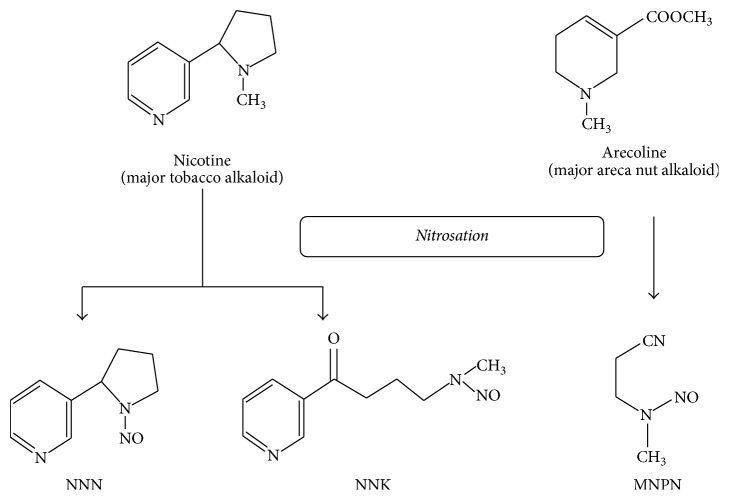
Major BQ alkaloids (arecoline) and major tobacco alkaloids (nicotine) can form nitrosamine substances by nitrosation reaction [17].

Some researchers reported that arecoline can produce nitrosamine through a nitrosation reaction in the digestive tract of the human body. Thus, BQ chewers swallowing BQ juice will increase their exposure to nitrosamine during the BQ chewing process [112]. The BQ chewing process will produce areca-specific nitrosamine substances that are known as N-nitrosoguvacine (NGC), N-nitrosoguvacoline (NGL), 3-methylnitrosaminopropionaldehyde (MNPA), and 3-methylnitrosaminopropionitrile (MNPN) (Figure 3) [16]. The most carcinogenic is MNPN, which is different from NNN and NNK during the nitrosamine reaction to chewing tobacco. MNPN can induce abnormal cell proliferation and carcinogenesis (Figure 4) [17].

Studies have shown that MNPN has a concentration of 0.5 to 11.4 *μ*g/L [113] in BQ chewers (excluding tobacco use). In addition, MNPN is carcinogenic in rat experiments. The IARC suggested that MNPN has sufficient evidence for carcinogenicity in experimental animals and is likely to be carcinogenic for humans (Group 2B) [9]. The carcinogenic capacity of MNPN may be due to easy formation of DNA methylation when MNPN is metabolized [98].

### 4.5. Molecular Mechanisms or Pathways Other Than Toxic Responses Involved in the Occurrence and Development of Oral and Pharyngeal Cancers

Numerous molecular mechanisms or pathways other than toxic responses, involving autophagy, hypoxia, COX-2, NF-*κ*B activity, and stemness are known to be induced by BQ ingredients and are very closely linked with the carcinogenesis of cancers of oral and pharynx. We have summarized these as follows.

Autophagy, a lysosomal degradation pathway, is an important process for cellular physiology and human health [114]. It is a pathogenesis modulator and potential treatment target for various diseases through regulation of pathogen elimination, apoptosis, immune system reaction, and cell development. When autophagy is activated or impaired, the oral cavity can be disturbed, because autophagy affects different functions and processes in the cavity, and the prognosis of the oral disease [115]. Autophagy plays dual function in the progress of oral and pharyngeal cancers [116]. Autophagy-induced tumor suppressor function is regulated by the removal of damaged oxidized organelles, which prevents the release of free radicals of oxygen that induce genomic instability [114]. In some studies, it is indicated that autophagy can boost tumor cells' survival during the development of cancer. Indeed, autophagy renders a protective function to limit the necrosis of tumor and inflammation and to lessen genome damage of tumor cells for metabolic stress or under poor nutrition conditions, particularly in solid and metastasizing tumors [115].

The upregulation of autophagy is likely to be the mechanism of cellular self-defense under stressful environmental conditions, which can protect tumor growth and may regulate tumor cells against therapy-induced apoptosis [115]. Treatment of oral cancer cells with ANE induced autophagy, which was identified through the accumulation of microtubule-related protein 1A/1B light chain 3-II (LC3-II) protein, the generation of autophagosomes, and the emergence of GFP-LC3 puncta. This action was regulated by the activation of p38, MKP-1, and hypoxia-inducible factor-1*α* (HIF1-*α*). The autophagy reaction was reduced by downregulation of ANE-modulated HIF1-*α* expression. The ANE-induced autophagy played a role in enhancing the portion of oral cancer cells undergoing the process of apoptotic death. It is clear that the ANE regulates a signaling cascade that causes the expression of HIF1-*α* in oral cancer cells. It was beneficial to the survival of cell from ANE-induced apoptosis in the eventual stimulation of autophagy [117]. The mechanisms of autophagy provide new insights into the pathogenesis of oral disorders and highlight their prominent roles in the development of oral carcinogenesis.

Hypoxia plays a critical role in oral cancers, pharyngeal cancers, and OPMD [118]. Cells under conditions of hypoxic stress may present many responses including increased angiogenic capacity, metabolic changes, and modified growth and survival of cells. The hallmarks of cancer consist of six biological events that happen in multiple separate steps in humans, namely, sustaining the proliferation of signaling, the resistance of cell death, evading growth suppressors, enabling replicative immortality, the induction of angiogenesis, and the activation of invasion and metastasis [119]. Risk factors (e.g., alcohol, BQ, and cigarette) use may activate the transcription of hypoxia responsive genes. HIF1-*α* is the major regulator of cellular responses to hypoxia [118]. The energy metabolism reprogramming and destruction of evading immune are emerging aspects of cancer that are significantly controlled by hypoxia-induced genes that mediate tumor angiogenesis, vascularization, invasion, metastasis, and drug resistance [118, 120]. In light of the multifactorial nature of tumor angiogenesis, reducing hypoxia is one of the possible approaches that could control the progress of oral and pharyngeal cancers. In future studies, it is crucial to highlight the hypoxia role in the carcinogenesis of cancers of oral and pharynx.

Regarding xenobiotic biotransformation, the cyclooxygenase-2 (COX-2) biological function is known not only to evoke the inflammation response but also to participate in the progress of cooxidation [121]. COX-2, a prostaglandin synthase, is responsible for the xenobiotic metabolism and inflammation response. In the progress of head and neck cancer, elevated expression of COX-2 is well known to have a critical role via some biological pathways [122–124]. ANE modulated the enhancement of mRNA and protein expression of COX-2, showing important roles in BQ chewing related oral mucosal disorders [42, 125]. A previous study indicated that BQ and its ingredients (arecoline/ANE) induced COX-2 expression* in vitro* [126]. Furthermore, the increased levels of COX-2 expression were found by using ANE and saliva-reacted ANE (sANE) treatments on three cell lines of oral epithelial carcinoma [19]. These results demonstrate an important insight into the potential effect of COX-2 and may contribute to the progress of BQ-related oral and pharyngeal cancers [19]. In BQ-related oral and pharyngeal cancers, upregulation of COX-2 can be promoted through a variety of upstream effectors. After a BQ/AN exposure, the upstream effectors may be released from the oral epithelial cells or oral microenvironment by linking to their corresponding sequences of COX-2 coding region or the promoter of COX-2 [19].

In proinflammatory and cancerous processes, the presence of two NF-*κ*B binding sites close to the promoter of COX-2 makes it very closely associated with NF-*κ*B activation [127, 128]. NF-*κ*B has been illustrated to be involved in the progress of tumorigenesis, and the expression of NF-*κ* parallels COX-2 expression in OPMD, as well as oral and pharyngeal cancers [124]. NF-*κ*B signaling pathways involve classic NF-*κ*B1 and alternative NF-*κ*B2 on the proinflammatory regulators induction and I*κ*B kinase complex activation [129]. Although the higher NF-*κ*B2 mRNA expression may indicate that the alternative pathway plays a more critical role in the upregulation of COX-2, both signaling pathways of NF-*κ*B may coexist in the BQ-induced activation of NF-*κ*B on oral epithelial cells [19]. The NF-*κ*B activation and ROS formation were induced by ANE and NF-*κ*B activation could be the foundation of the occurrence of ROS [117]. After treating ANE in OECM-1 and SAS oral keratinocytes, NF-*κ*B and the mitogen-activated protein kinases activation have been indicated [130]. Also, ANE treatment may enhance the upregulation of COX-2 and NF-*κ*B within normal human oral keratinocyte and is likely to associate with G1/S phase arrest of cell cycle and occurrence of cellular senescence [131]. Areca nut-derived arecoline can induce the expression of alphavbeta6 (*α*v*β*6) integrin via the muscarinic acetylcholine receptor M(4) in oral keratinocyte [132].

Solid tumors are encircled by a specific tumor microenvironment that combines blood and lymphatic vessels, extracellular matrix, and mesenchymal and immune cells. The tumor microenvironment conducts many of the tumor aggression features, such as local metastasis and invasion. In tumor cells, tumor microenvironment can induce stem cell-like programs to form cancer stem cells which are known as cancer “stemness” [133]. Recent evidence indicates that cancer stem cells are responsible for the recurrence of tumors and are resistant to current common modes of treatment for cancer. A review article suggested that COX-2 triggers cancer stemness and support the maintenance of stem cells [134].

Overall, in oral cancer cells, ANE can induce oxidative stress, such as ROS, and upregulate hypoxia inducing factors leading to autophagy [117]. Although oral and pharyngeal cancers can be prevented and discovered early, the overall survival rate remains only about 50%. Most oral and pharyngeal cancers may be formed through the progress of clinical lesions named OPMD. It is difficult to predict when the lesions transform into malignant ones and to provide appropriate strategies for their management. Understanding the molecular pathways involved will help us to prevent malignant transformation of OPMD and find useful strategies for early diagnosis and prevention of oral and pharyngeal cancers.

### 4.6. BQ Abuse/Addiction

BQ chewing has longstanding cultural and recreational importance in Asian and South Pacific communities. It is used in religious ceremonies and gatherings and forms a part of daily life in these regions [3, 135]. Because of cultural traditions, its use is socially acceptable among all socioeconomic groups, even in women and young children [5, 136]. These circumstances favor a sociocultural niche conducive for people to use and abuse this substance. However, to our knowledge, population data regarding BQ abuse and dependent use has not been available for the regions of Asia. Recently, the intercountry survey was initiated by BQ research groups of east, southeast, and southern Asia to investigate these issues [48]. Furthermore, tolerance and withdrawal syndromes to BQ were distinguished in habitual chewers [8, 135, 137]. Such neurobiological characteristics are analogous to those attributed to tobacco, a major psychoactive substance that can lead to misuse and dependence.

Currently, there are no accredited criteria for “BQ-mania” that lead to clinically significant BQ addiction-related disorders. The groups of Asian BQ consortium have developed a validated BQ addiction screening tool and scoring approach, and this effort elucidated the psychiatric aspects of BQ dependency (BQ-D) by understanding its sociodemographic, substance use, and environmental approachable heterogeneity undermining its population burden and health effects. In intercountry survey, the Taiwanese study revealed that the prevalence rates of BQ abuse in men (4.9%) were higher than in women (1.7%) [13]. In addition, we found a 46.0% BQ abuse rate in men current-chewers and a 68.8% BQ abuse rate in women current-chewers in Taiwan using a validated BQ-abuser screening tool and scoring approach [13].

In an intercountry survey in Taiwan, the prevalence of BQ-D, defined independently through the criteria of the Diagnostic and Statistical Manual of Mental Disorders, Fourth Edition (DSM-IV), and International Statistical Classification of Diseases and Related Health Problems, 10th Revision (ICD-10), was higher among men (3.5%–4.2%) than among women (1.1%), and the dependence rate in chewers was 41.7% for male chewers and 45.1% for female chewers [14]. One Indian study applied the criteria from DSM-IV-TR (Text Revision) to diagnose a substance use disorder along with the ICD-10 criteria for psychoactive substance use to define BQ-D and found that AN with or without tobacco are associated with the BQ-D syndrome development [135]. Our previous study suggested that alcohol usage was strongly related to BQ chewing in Taiwan aborigines [43]. To determine behavioral predictors of BQ chewing, 7144 aborigines were recruited into a community-based study in Taiwan. Habitual alcohol drinking was a significant factor associated with cessation of BQ use, while smoking had no statistical association with chewing BQ. Hence, efforts to abstain from alcohol might be effective in trying to quit the habit of BQ chewing [43].

### 4.7. The Mechanism for BQ Abuse/Addiction and Related Disorders* In Vivo* and* In Vitro*

BQ addiction and related disorders include BQ abuse, BQ dependence, and BQ abuse combined with BQ dependence. It is evident that BQ addiction has roots in complex behaviors; however, its precise biology is unknown. In rat brains, the dichloromethane fraction extracted from the areca palm* (Areca catechu)* of AN seed has antidepressant properties due to its inhibition of monoamine oxidase A (MAO-A) [138], but this is a very minor component of BQ and it is unknown whether it is consumed sufficiently among the styles of BQ chewing to affect the user. It is certain that the principle component of the AN is arecoline that passes through the blood-brain barrier [139, 140] and *γ*-amino-butyric acid (GABA) is a competitive inhibitor as well as an agonist to acetylcholine muscarinic receptors. As such, it has been reported to enhance acetylcholine levels in animal brains [141, 142] and inhibit the expression of MAO-A in male albino rats [143]. Thus, in the pathway of dopamine-rich mesolimbic reward, it remains unclear whether arecoline could have a putative role by way of directly inhibiting MAO-A or using acetylcholine as a proxy or inhibit GABAergic interneurons to enhance addiction similar to that in tobacco smoking and alcoholism [144].

Numerous susceptible genes are implicated in the mechanisms of addiction, and the optimal approach to selecting candidate genes is complicated. In spite of this, our study has hinted at the significance of the MAO-A gene (Xp 11.3) in heavy BQ use [145]. MAO catalyzes the deamination of biogenic amines, thus regulating synaptic levels of dopamine, serotonin, norepinephrine, and catecholaminergic neurotransmitters [144] to influence addictive behaviors, motor, memory, and mood [146]. Because MAO-A has been involved in the dopaminergic tone modulation to facilitate reinforcing behaviors [147], this provides a likely addiction framework for BQ chewing. In animal brains, arecoline has been found to enhance cortical dopamine. In these studies, 15 minutes after the administration of arecoline, a reduction in the levels of acetylcholine and norepinephrine was observed [148]. Recently, study findings suggested increased dopamine levels are likely derived, at least in part, from MAO-A inhibition [145]. Additionally, the amount of 6 different neurotransmitters was determined in rat brains using LC-MS-MS [149].

Our recent results showed that direct treatment of neuroblastoma cells with ANE and arecoline progressively inhibited MAO-A in a dose-dependent manner; this was measured via microarray and confirmed by qRT-PCR and* in vivo* rat studies [145]. In experimental animals, conditioned place preference (CPP) is one of the most common methods to evaluate nondrug therapy and the motivational effects of drugs and to quantify drug reward in laboratory animals [150]. The CPP model is conducted to explore the reinforcing properties of drug abuse and maladaptive behaviors progress. In a previous study, the CPP paradigm was used to observe the role of candidate genes in the progress of substance-induced CPP [151].

In human psychopharmacology researches, positron emission tomography (PET) and single photon emission computed tomography (SPECT) have been designed as increasingly effective imaging tools during the last two decades. PET and SPECT have been established to enhance pharmacokinetics and pharmacodynamics of substance addiction and have created many contributions in terms of addiction mechanisms [152]. In the [11C] befloxatone study, PET indicated that the cerebral MAO-A inhibition caused by components of cigarette smoke could be involved in tobacco addiction [153]. The role of the striatal dopamine system has been thought to encompass circuits that are involved in the coordination of motor [154]. A recent study has involved this system in the responses of reward and cognitive activities integration and by the human corticostriatothalamic systems [155]. Dopamine transporter (DAT) is one of the crucial presynaptic factors complicated in modulating dopaminergic tone. Some imaging studies also reported that availability of striatal DAT was prominently related to the functional impact of mood regulation, various cognitive actions, and complicated social behavior by using SPECT with [99mTc] TRODAT-1 (a radio-labeled form of tropane derivative for the selective labeling of DAT) [156–158].

### 4.8. MAO-A Variants Associated with Increased BQ Use

In Taiwan aborigines, our previous results provided a novel finding that variants of MAO-A were significantly related to the behaviors of heavy BQ consumption. Genetic polymorphism of MAO-A single-nucleotide polymorphisms (SNPs) in both men (OR = 2.04 for rs2283725; OR = 2.03 for rs5953210) and women (OR = 1.54 for rs2283725; OR = 1.59 for rs5953210) appeared to correlate with a higher likelihood of heavy BQ chewing. Compared with nonusers, the MAO-A activity was significantly higher among BQ users. This effect was enhanced strongly with alcohol use. Subjects with specific* MAO-A* polymorphisms that have a higher enzyme activity of MAO-A predispose to BQ abuse liability among Taiwan aborigines [145].

### 4.9. BQ Abuse-Related MAO Variants May Be Related to the Risk of BQ-Associated Oral and Pharyngeal Cancers

The occurrence of oral and pharyngeal cancers is implicated in a joint effect between exposure of environmental factors (e.g., alcohol, BQ, and cigarette) and gene expression. A microarray study conducted by our group found that BQ and its components (ANE/arecoline) induced the expression of addiction-related MAO genes* in vitro*. Our previous studies suggested that MAO-A and monoamine oxidase B (MAO-B) variants are associated with oral and pharyngeal cancer risks [159]. We confirmed that downregulation of MAO-A and MAO-B gene was more prominent in oral and pharyngeal cancerous tissues than in their adjacent noncancerous tissues. Moreover, we demonstrated that BQ chewing and MAO-A polymorphisms are linked with oral and pharyngeal cancers [145]. The single-nucleotide polymorphism variants of MAO-A were significantly related to patients with oral and pharyngeal cancers in comparison to patients with OPMD (risk G-allele for rs5953210, OR = 1.76; 95% CI = 1.02–3.01) [145]. Therefore, these results provide a crucial insight into the potential impact of MAO variants in contributing to the occurrence of BQ-related oral and pharyngeal cancers.

The above results will provide new insights into the behavior of BQ use and related oral and pharyngeal cancers and other disorders. The strong implication of high BQ exposure in the development of BQ addiction and related disorders indicates that our country must become more aware of this issue. As is illustrated above, an association of BQ exposure with BQ-related disorders has been shown in humans. Studies of complex traits such as the occurrence of oral and pharyngeal cancers should incorporate both genetic and environmental factors. In the future, we must study the association between BQ-related disorders and the expression of two susceptibility genes* (MAO-A*/*MAO-B)* related to BQ pathways or neurotransmitter metabolism. Alkaloids, the active metabolic compounds of BQ, can interact with neurotransmitters and susceptibility genes to exacerbate BQ-related disorders.

### 4.10. The Draft of BQ Withdrawal Policy with Taiwan as an Example

In the Ministry of Health and Welfare (MOHW), Taiwan, the buzzword is “ABC 123” for health education indicating that the synergistic joint effects of alcohol (A), BQ (B), and cigarette (C) enhance 123-fold risk for the occurrence of oral cancer. The 123-fold risk was obtained from a highly cited paper [10], and this paper has already been cited more than 440 times [10]. According to these results, MOHW established “the prevention day of BQ” in December (12), 3, every year. These efforts promoted the management policy of our government and help to enhance the strategy of “BQ Education.” At present, the BQ researchers and epidemiologists have become prominent in Taiwan. BQ chewing is strongly related to alcohol drinking and/or smoking, and preventive policies should be concentrated on the cessation pattern of BQ chewing and the related individual habits of alcohol and tobacco use.

The area of human health effects of group 1 carcinogens, such as BQ, has been identified as an important research topic in Taiwan. Future research should aim to fill gaps in our knowledge concerning the effect of BQ abuse/dependent use on health consequences in Taiwanese communities. BQ addiction and the associated high prevalence of oral and pharyngeal cancers in Taiwan also highlight the uniqueness and health relevance of this review article. Our efforts can provide an effective methodology to reduce the rates of BQ chewing. Therefore, these effects have enhanced the management policy of our government and helped to promote the strategy of “Outpatient Services for BQ Screening and Cessation.”

A decreasing amount of BQ consumption is being observed in Taiwan after continuing the strategies of successive health promotion. In 2008, the Bureau of Health Promotion of MOHW assigned a BQ cessation plan to us for exploring specific exposure indicators among chewers and entitled this project “specific biomarkers of chewing behavior and cessation patterns among BQ chewers” [15]. Recently, an intercountry study indicated that Taiwanese men have a higher quit rate (31.1%) than other countries, and this may be a result of effective BQ prevention activities and interventions [48]. In conclusion, we believe that these endeavors may offer effective strategies of public health against BQ chewing related health effects.

## 5. Conclusion

This review provides important insights regarding the potential role of environmental BQ in the occurrence and progress of oral and pharyngeal cancers and related risks of human health. Subsequent molecular mechanisms and pharmacokinetics studies will establish a stable foundation for the prevention, clinical diagnosis, and treatment effectiveness of BQ addiction-related disorders.

## Figures and Tables

**Table 1:** Epidemiology studies of BQ usage related multidimensional health effects.

Health effects	Location (year)	Study design	Number of cases	Number (%)^a^ of BQ	Number of controls	Number (%) of BQ	aOR (95% CI)	Ref.
Oral cancer	Kaohsiung, Taiwan (1992-93)	Case-control study (*N* = 307)	107 (M/F = 104/3)	Never: 31 (29%) Former: 5 (5%) Current: 71 (66%)	200 (M/F = 194/6)	Never: 153 (77%) Former: 5 (3%) Current: 42 (21%)	BQ only: 28.2 (1.9–414.4)^*∗*^ A + BQ + C: 122.8 (171.1–880.5)^*∗*^	[10]

Pharyngeal cancer	Kaohsiung, Taiwan (2000–03)	Case-control study (*n* = 501): frequency matched males >= 40 yr	148 (M = 148)	Never: 33 (22%) Former: 44 (30%) Current: 71 (48%)	255 (M = 255)	Never: 216 (85%) Former: 11 (4%) Current: 28 (11%)	7.7 (4.1–15.0)^*∗*^	

Oral leukoplakia	Kaohsiung, Taiwan (1994-95)	Case-control study (*N* = 1095)	125 (M/F = 118/7)	Never: 19 (15%) Former: 6 (5%) Current: 100 (80%)	500 (M/F = 472/28)	Never: 258 (52%) Former: 16 (3%) Current: 226 (45%)	22.3 (11.3–43.8)^*∗*^	[18]

Oral submucous fibrosis			94 (M/F = 93/1)	Never: 10 (11%) Former: 5 (5%) Current: 79 (84%)	376 (M/F = 372/4)	Never: 188 (50%) Former: 14 (4%) Current: 174 (46%)	40.7 (16.0–103.7)^*∗*^	

Laryngeal cancer	Kaohsiung, Taiwan (2000–03)	Case-control study (*n* = 501): frequency matched males >= 40 yr	128 (M = 128)	Never: 85 (66%) Former: 15 (12%) Current: 28 (22%)	255		1.3 (0.7–2.5)	[12]

Esophageal cancer	Kaohsiung, Taiwan (2005)	Case-control study (*N* = 420): age, sex matched males (>= 40 yr)	165 (M = 165)	Never: 72 (44%) Former: 31 (19%) Current: 62 (38%)	255 (M = 255)	Never: 216 (85%) Former: 11 (4%) Current: 28 (11%)	1.7 (0.8–3.1) Chewed with a piece of betel inflorescence: 4.2 (1.4–16.0)^*∗*^ Swallowed BQ juice: 3.3 (1.3–9.3)^*∗*^	[21]

Hepatocellular carcinoma (HCC)	Kaohsiung, Taiwan (1996-97)	Case-control study (*N* = 526): matched by sex and age (±3 years)	263 (M/F = 205/58)	Never: 192 (73%) Ever: 71 (27%)	263 (M/F = 205/58)	Never: 241 (92%) Ever: 22 (8%)	3.49 (1.74–6.96)^*∗*^	[22]

Hepatocellular carcinoma (HCC)	Taiwan (2003)	Community-based cohort study	11837 (M = 11837)	Never: 10,388 (88%) Ever: 1449 (12%)			Compared with non-BQ chewers with HBsAg(−): BQ chewers with HBsAg(−): 3.43 (1.19–9.89)	[41]

Cirrhosis	Kaohsiung, Taiwan (1996-97)		210 (M/F = 170/40)	Never: 176 (84%) Ever: 34 (16%)	210 (M/F = 170/40)	Never: 199 (95%) Ever: 11 (5%)	3.56 (1.41–8.96)^*∗*^	[25]

HCC complicating cirrhosis	Kaohsiung, Taiwan (1996-97)	Case-control study (*N* = 630): matched by sex and age (±5 yr)						[23]
Cirrhosis with HCC			210 (M/F = 170/40)	Never: 158 (75%) Ever: 52 (25%)	210 (M/F = 170/40)	Never: 199 (95%) Ever: 11 (5%)	Compared with controls: 5.81 (2.26–14.94)^*∗*^	
							Compared with cirrhosis alone: 1.69 (1.04–2.76)^*∗*^	

Adverse birth outcomes	East, Taiwan (1998)	Case-control study (*N* = 229)	F = 32	Never: 10 (31%) Ever: 22 (69%)	F = 197	Never: 101 (51%) Ever: 96 (49%)	Low birth weight: 9.1 (1.6–51.8)^*∗*^ Preterm birth: 3.4 (1.1–11.4)^*∗*^	[38]

Adverse pregnancy outcomes	Taiwan Bunum aborigine (1994)	Cross-sectional study: DOH-HR survey (age-matched) (*N* = 186)	Adverse pregnancy: 62 (F = 62) Never-adverse pregnancy: 124 (F = 124)	Never: 35 (56.4%) Ever: 27 (43.6%)			Adverse pregnancy outcomes: 2.8 (1.2–6.8)^*∗*^	[42]

Lower birth weight (LBW)	South and East, Taiwan (2003-04)	Cross-sectional study: hospital-based (*N* = 1264)	1264	Never: 800 (63%) Ever: 464 (37%)			LBW: 2.40 (1.21–4.80)	[37]

Full-term LBW							Full-term LBW: 3.67 (1.70–7.96)	

Lower male newborn rate							Male newborn: 0.62 (0.43–0.89)	

Asthma	Kaohsiung, Taiwan (2013)	Case-control study (*N* = 1800) (age- and gender-matched community controls)	600	M: Never: 192 (75%) Ever: 63 (25%) F: Never: 340 (99%) Ever: 5 (1%)	1200	M: Never: 426 (84%) Ever: 84 (16%) F: Never: 689 (99.9%) Ever: 1 (0.1%)	Current chewers: 2.05 (1.12–3.76)^*∗*^	[40]

Metabolic syndrome	Taiwan (1993–96)	Cross-sectional study: nutrition and health survey (NAHSIT) (*N* = 1986)	1986 (M/F = 920/1066)	M: Never: 634 (69%) Ever: 286 (31%) F: Never: 984 (92%) Ever: 82 (8%)			Metabolic syndrome: 1.31 (1.12–1.55)^*∗*^ (BQ consumption 10 times/day) Abdominal obesity: 1.42 (1.2–1.68)^*∗*^ Hypertriacylglycerolemia: 1.33 (1.02–1.73)^*∗*^ High blood pressure: 2.0 (1.4–3.0)^*∗*^ (only in females)	[27]

Metabolic syndrome	Keelung, Taiwan (2001–03)	Cross-sectional study: Keelung community-based integrated screening (KCIS) program (*N* = 19866)	19866 (M = 19866)	M: Never: 16874 (85%) Former: 1569 (8%) Current: 1423 (7%)			Former: Metabolic syndrome: 1.38 (1.19–1.61)^*∗*^ Hyperglycemia: 1.07 (0.87–1.31) Hypertriacylglycerolemia: 1.40 (1.23–1.61)^*∗*^ Current: Metabolic syndrome: 1.78 (1.53–2.08)^*∗*^ Hyperglycemia: 1.24 (1.09–1.64)^*∗*^ Hypertriacylglycerolemia: 1.90 (1.66–2.19)^*∗*^	[26]

Type 2 DM	Keelung, Taiwan (1999–2001)	Cross-sectional study: KCIS program (*N* = 14816)	14816 (M = 14816)	M: Never: 12696 (86%) Ever: 2120 (14%)			Type 2 DM: 1.29 (1.04–1.60)^*∗*^	[28]

Obesity	Taiwan aborigines (2003-04)	Cross-sectional study: large-scale survey of substance use (*N* = 7144)	7144 (M/F = 3824/3320)	M: Never: 1791 (47%) Former: 167 (4%) Current: 1866 (49%) F: Never: 2062 (62%) Former: 84 (3%) Current: 1174 (35%)			Obesity: 1.61 (1.40–1.85)^*∗*^	[43]

Heart disease	Taiwan (1993–96)	Cross-sectional study: nutrition and health survey (*N* = 1932) (NAHSIT)	1932 M/F = 896/1036	M: Never: 619 (69%) Ever: 277 (31%) F: Never: 957 (92%) Ever: 79 (8%)			Heart disease: 1.37 (1.1–1.6)^*∗*^ (BQ consumption 10 times/day; only in females)	[32]

Cardiovascular disease (CVD) All-cause mortality	Taiwan (1998-99)	Cross-sectional study: MJ health screening centers (*N* = 56116) follow-up study	56116 (M = 56116)	Never: 44565 (79%) Former: 5568 (10%) Current: 5983 (11%)			Former: CVD: 1.56 (1.02–2.38) All-cause mortality: 1.40 (1.17–1.68) Current: CVD: 2.02 (1.31, 3.13) All-cause mortality: 1.40 (1.16, 1.70)	[30]

Cardiovascular disease (CVD)	Keelung, Taiwan (1999–2004)	Cross-sectional study: KCIS program (*N* = 21906)	21906 (M = 21906)	Never: 17976 (82%) Former: 1830 (8%) Current: 2100 (10%)			Ever: CVD: 1.24 (1.11–1.39)^*∗*^	[31]

Cardiovascular disease (CVD)	New Taipei, (2013)	Cross-sectional study (hospital-based)	3177 (M/F = 2002/1175)	M: Never: 1761 (88%) Ever: 241 (12%) F: Never: 1167 (99.3%) Ever: 8 (0.7%)			Ever: CVD risk factors: Obesity: 1.43 (1.07–1.91) Central obesity: 2.27 (1.53–3.37) hs-CRP level: 1.38 (1.03–1.85)	[44]

Obesity metabolic syndrome Type 2 diabetes mellitus (DM) Cardiovascular disease (CVD) All-cause mortality	17 Asia studies (5 cohort, and 12 case-control studies) (1951–2013)	Meta-analysis (*N* = 121585)	Obesity (*N* = 30623) metabolic syndrome (*N* = 23291) DM (*N* = 51412) CVD (*N* = 201488) All-cause mortality (*N* = 179582) Hypertension (*N* = 89051)				Obesity: 1.47 (1.23–1.75)^*∗*^ Metabolic syndrome: 1.51 (1.09–2.10)^*∗*^ Type 2 DM: 1.47 (1.20–1.81)^*∗*^ CVD: 1.2 (1.03–1.40)^*∗*^ All-cause mortality: 1.21 (1.04–1.42)^*∗*^ Hypertension: 1.45 (0.98–2.15)	[45]

All causes Cancers	Araihazar, Bangladesh (2000–02)	Cohort study (*N* = 19999)	19999 (M/F = 8148/11851)	M: Never: 5010 (61%) Former: 288 (4%) Current: 2850 (35%) F: Never: 7989 (67%) Former: 177 (2%) Current: 3685 (31%)			Ever chewers: All causes: 1.26 (1.09–1.44)^*∗*^ Cancers: 1.55 (1.09–2.22)^*∗*^ Cardiovascular disease: 1.16 (0.93–1.43)	[34]

Total death Cerebrovascular deaths	Taiwan (1989–96)	Cohort study (*N* = 6503)	6503 (M/F = 3577/2926)	Never: 5602 (44%) Former: 373 (19%) Current: 528 (38%)			Total death: 1.19 (1.05–1.35) Cerebrovascular deaths: 1.66 (1.19–2.30)	[35]

Schizophrenia	Palau (1998)	Cross-sectional study: hospital-based study (*N* = 70)	70 (M/F = 49/21)	M: Never: 26 (53%) Ever: 23 (47%) F: Never: 4 (19%) Ever: 17 (81%)			BQ chewing associated with milder symptom schizophrenia	[46]

Schizophrenia	Palau (2002–04)	Cohort study: (*N* = 65)	65 (M/F = 45/20)	Never: 16 (25%) Ever: 49 (75%)			Male BQ chewers with high-consumption had significantly milder positive symptoms than BQ chewers with low-consumption	[47]

^a^May not total 100% due to rounding. A: alcohol drinking; B: betel quid chewing; C: cigarette smoking; never: never-chewers; former: former chewers (withdraw BQ); current: current chewers; ever: ever chewers (former chewers combined current chewers); ref.: reference; DM: diabetes mellitus; hs-CRP: high-sensitivity C-reactive protein; CVD: cardiovascular disease. *∗* indicates a statistically significant difference (*p* < 0.05).

**Table 2:** A summary table showing data for prevalence of BQ chewing among adults and their incidence and mortality of oral and pharyngeal cancers available from various countries.

Country, publication year [Ref]^d^	Lifetime prevalence of chewing^a^			Incidence^b^ of oral and pharyngeal cancers^c^						Mortality^b^ of oral and pharyngeal cancers					
	Males	Females	Total	Males			Females			Males			Females		
	(%)	(%)	(%)	O^e^	P^f^	O/P^g^	O	P	O/P	O	P	O/P	O	P	O/P
World	—^h^	—	10.0	5.5	3.2	8.7	2.5	0.7	3.2	2.7	2.2	4.9	1.2	0.5	1.7
*South-Eastern Asia*	—	—	—	4.0	2.6	6.6	2.5	0.7	3.2	1.9	2.1	4.0	1.2	0.5	1.7
Indonesia, 2011 [48]	12.4	47.8	30.2	2.8	1.4	4.2	1.9	0.5	2.4	1.2	1.1	2.3	0.8	0.4	1.2
Thailand, 1987 [49]	16.0	19.0	17.0	5.1	2.6	7.7	3.0	0.4	3.4	2.6	1.6	4.2	1.6	0.3	1.9
2012 [50]	0.3	3.3	1.8^i^												
Myanmar, 2006 [51]			24.5	8.6	9.1	17.7	4.1	1.7	5.8	5.3	8.3	14.6	2.5	1.5	4.0
2016 [52]	72.4	38.6	52.4												
Philippines —	—	—	—	4.1	2.6	6.7	3.2	1.2	4.4	1.7	2.2	3.9	1.6	1.3	2.9
Vietnam, 2008 [53]	—	6.7	—	3.3	2.6	5.9	1.6	0.5	2.1	1.5	2.2	3.7	0.7	0.4	1.1
Malaysia, 1997 (Sarawak) [54]	30.3	63.5	49.3	3.3	1.7	5.0	2.8	0.6	3.4	1.2	0.8	2.0	0.8	0.3	1.1
1998 (Sarawak) [55]	25.2	61.2	45.9												
2000 [56]	23.7	76.3	18.4												
2011 [57]	4.8	10.5	8.2												
2011 [48]	10.3	32.1	23.8												
Cambodia, 1995 [58]	6.8	40.6	31.2	7.1	3.8	10.9	5.2	1.1	6.3	4.1	3.4	7.5	2.9	1.0	3.9
2016 [59]		19.7													
Singapore —	—	—	—	3.4	2.0	5.4	1.7	0.3	2.0	1.1	0.7	1.8	0.4	0.1	0.5
*South-Central Asia*	—	—	—	9.9	6.2	16.1	4.7	1.4	6.1	6.3	5.3	11.6	3.0	1.2	4.2
India, 1996 [60]	37.8	29.7	33.0	10.1	6.3	16.4	4.3	1.3	5.6	6.7	5.3	12.0	3.0	1.1	4.1
2015 [61]															
BQ with tobacco	7.5	4.9	6.2												
*Gutka* & other areca nut mixtures	13.1	2.9	8.2												
*Pan masala or BQ without tobacco*	3.5	5.4	4.4												
Bangladesh, 2015 [29]	38.4	32.5	34.9	13.0	14.9	27.9	5.9	3.1	9.0	7.7	12.9	20.6	3.5	2.7	6.2
Pakistan, 1974 [62]	27.9	37.8	32.5	10.5	3.0	13.5	9.1	4.1	13.2	6.3	2.6	8.9	5.4	1.3	6.7
2006 [63]			37.8												
2007 [64]			40.0												
2008 [65]	34	31	32												
Sri Lanka, 1992 [66]	54.0	42.0	45.2												
2011 [48]	21.2	14.5	16.9	15.5	7.2	22.7	5.7	3.3	9.0	5.2	5.2	10.4	1.9	2.3	4.2
Nepal, 2011 [48]	43.6	34.9	40.7	7.2	3.9	11.1	2.1	1.0	3.1	4.8	3.5	8.3	1.4	0.9	2.3
*Eastern Asia*	—	—	—	2.4	1.3	3.7	1.1	0.2	1.3	1.1	0.7	1.8	0.5	0.1	0.6
Taiwan, 1992 [67]	16.5	2.9	10.0	27.7	14.0	41.7^j^	2.7	0.8	3.5^j^	—	—	15.1^j^	—	—	1.2^j^
2005 [68]	18.9	1.7													
2005 [69]	14.5	0.1	7.2												
2008 [70]	20.9	1.2													
2011 [48]	15.6	3.0	8.9												
Mainland China, 1997 (Hunan) [71]	39.3	30.5	35.4	1.6	0.6	2.2	0.9	0.1	1.0	0.9	0.4	1.3	0.4	0.1	0.5
2011 (Hunan) [48]	29.0	2.3	16.2												
Japan —	—	—	—	3.9	3.5	7.4	2.0	0.4	2.4	1.0	1.2	2.2	0.7	0.2	0.9
Republic of Korea —	—	—	—	2.9	2.4	5.3	1.6	0.2	1.8	1.0	1.1	2.1	0.4	0.1	0.5
*Pacific islands*															
Papua New Guinea, 1968 [72]	62.8	52.8	57.7	30.3	4.5	34.8	21.2	0.5	21.7	19.4	3.6	23.0	10.3	0.4	10.7
2007 [73]			76												
2008 [74]			93.1												
Solomon Islands, 2007 [4]	83.0	68.4	76.8	5.7	0.8	6.5	1.5	0.0	1.5	3.9	0.8	4.7	0.8	0.0	0.8
Palau, 1996 [75]	72.0	80.0	—												
Guam —	—	—	—	5.2	0.0	5.2	0.0	0.0	0	2.0	0.0	2.0	0.0	0.0	0.0

^a^from IARC report [9]; ^b^GLOBOCAN 2012 [76]; ^c^the age-standardized rates per 100,000 (standardized to the world standard population); ^d^[Ref]: reference; ^e^O: oral cancer; ^f^P: pharyngeal cancer; ^g^O/P: oral and pharyngeal cancers; ^h^—: no recent data were available; ^i^BQ with tobacco; ^j^from Ministry of Health and Welfare, Cancer Registration System Annual Report, Taiwan, 2012 [77].
